# The impact of Swanson’s theory of caring on self-efficacy and pregnancy outcomes of primiparous women

**DOI:** 10.12669/pjms.41.11.12959

**Published:** 2025-11

**Authors:** Caifang Zhu, Yuying Zhang

**Affiliations:** 1 Caifang Zhu Department of Obstetrics and Gynecology, uzhou Hospital of Integrated Traditional Chinese and Western Medicine, Suzhou, Jiangsu Province 215101, P.R. China; 2 Yuying Zhang Party and Government Affairs Office, uzhou Hospital of Integrated Traditional Chinese and Western Medicine, Suzhou, Jiangsu Province 215101, P.R. China

**Keywords:** Swanson’s theory, Self-efficacy, Pregnancy outcomes, Primiparous women

## Abstract

**Objective:**

To explore the impact of Swanson’s theory of caring on self-efficacy and pregnancy outcomes in primiparous women.

**Methodology:**

his single-center, retrospective case-control analysis included 130 primiparous women who underwent prenatal check-ups and completed delivery at Suzhou Hospital of Integrated Traditional Chinese and Western Medicine between March 2023 to March 2025. According to the prenatal care received from 28 to 37 weeks of pregnancy, participants were divided into the Swanson group (based on Swanson’s theory of caring in nursing) and the control group (routine nursing). Two groups were matched in a 1:1 ratio, with 65 cases in each group. The primary outcomes were childbirth self-efficacy and childbirth coping ability. The secondary outcomes included the degree of pain during labor, assessed using the Visual Analog Scale (VAS), the total duration of labor, the 1-minute Apgar score of the newborn and pregnancy outcomes.

**Results:**

After intervention, the scores of Self-Efficacy Expectations (EE-16), Outcome Expectations (OE-16) and the Chinese version of the Childbirth Self-Efficacy Inventory (CBSEI-C32) in both groups significantly increased compared to before intervention and were considerably higher in the Swanson group compared to the control group (P<0.05). The delivery VAS, total labor duration, cesarean section rate and incidence of adverse pregnancy outcomes in the Swanson group were significantly lower than those in the control group. Swanson’s theory of caring was associated with a considerably higher one-minute Apgar score than routine nursing (P < 0.05).

**Conclusions:**

Interventions based on Swanson’s care theory can effectively improve the self-efficacy of primiparous women during labor, reduce pain levels, shorten labor duration, promote vaginal delivery and improve maternal and infant outcomes.

## INTRODUCTION

Psychological stress is a common problem among postpartum women, especially first-time mothers.^1^-^4^ Due to the lack of childbirth experience and the pain caused during delivery, primiparous women are prone to negative emotions such as anxiety and depression and are losing confidence and expectations in vaginal delivery, which leads to an increase in the rate of cesarean sections.^3^-^5^ Studies show that pregnancy-related anxiety also increases the risk of adverse pregnancy outcomes such as premature birth and postpartum hemorrhage.^5^,^6^ In addition, the lack of knowledge about prenatal care, delivery mode selection, postpartum care, etc., among primiparous women often impacts their self-managing abilities during pregnancy.^4^-^6^ Furthermore, first-time mothers are prone to emotional fluctuations and changes in their neuroendocrine system during the delivery process, which can increase the incidence of adverse outcomes for both mother and offspring.^5^-^7^

Self-efficacy refers to the degree of confidence that individuals have in their ability to use the skills they possess to complete a specific work behavior.^8^,^9^ In recent years, studies have confirmed that nursing interventions based on self-efficacy as a theoretical framework can improve patients’ self-management efficacy levels, behavioral changes and health outcomes.^8^-^10^ A meta-analysis by Cho et al.^8^ showed that such interventions can significantly improve self-efficacy and treatment compliance in patients with obstructive sleep apnea. A study by Bień et al.^9^ reported the importance of self-efficacy during pregnancy. A randomized controlled trial by Ip WY et al.^10^ demonstrated that prenatal health education can improve self-efficacy and the ability to cope with childbirth, alleviate pain and anxiety during labor.

Various nursing interventions are designed to improve the self-efficacy of primiparous women and enhance maternal and infant outcomes. Abdelaziz et al.^11^ showed the efficiency of the Internet-based cognitive behavioral therapy program in significantly reducing fertility phobia and strengthening the sense of self-efficacy of pregnant women, supporting the integration of the platform into perinatal care. Continuous support and education have been shown to reduce the fear of childbirth and improve self-efficacy in primiparous women.^12^ However, a meta-analysis of 31 studies^13^ found that while prenatal education can improve the psychological outcomes of mothers and promote vaginal delivery, the comparative effectiveness of various educational methods is still unclear. Recent literature has emphasized the effectiveness of individualized psychological and educational interventions for primiparous women. For instance, Dai et al.^14^ reported that simulation-based childbirth education significantly improved the psychological readiness of Chinese primiparas and enhanced childbirth confidence. Alizadeh-Dibazari et al.^15^ demonstrated that higher levels of perceived social support were associated with reduced fear of childbirth. Similarly, a recent meta-synthesis by Lunda et al.^16^ concluded that continuous emotional support during labor improved self-efficacy and reduced anxiety.

Moreover, Frankham et al. found that online childbirth education reduced postpartum PTSD symptoms and improved maternal self-efficacy.^17^ Compared to interventions such as mindfulness training and cognitive-behavioral therapy, Swanson’s theory of caring offers a broader, personalized care framework integrating emotional presence, empowerment, and belief maintenance. This structured approach may yield greater psychological benefits in a population such as primiparous women, who are particularly vulnerable to anxiety and uncertainty during childbirth. Delivery guidance focuses on the technical aspect of delivery, neglecting the emotional needs and psychological experiences of the mother.^11^-^13^

The Swanson’s theory of care was proposed by American nursing expert Kristen Swanson. It includes five care processes: knowing, being with, doing for, enabling and maintaining belief.^18^ The effectiveness of nursing interventions based on Swanson’s theory of care has been confirmed in multiple disciplines. Lee et al.^19^ demonstrated the effectiveness of this approach in helping families of children with rare diseases gain positive beliefs and achieve meaningful lives. Palas Karaca et al.^20^ found that personalized care based on Swanson’s theory of care has a positive impact on the levels of sadness, depression, anxiety and stress in women who have experienced miscarriage. Recent studies from Pakistan have also highlighted the significance of psychosocial interventions in improving maternal health outcomes, particularly in low-resource settings.^21^-^24^ However, there is still a lack of structured, theory-based nursing interventions focused on primiparous women’s self-efficacy within South Asian healthcare systems, which this study seeks to address. However, the research on the effects of this method on self-efficacy and pregnancy outcomes, especially for primiparous women, is still scarce. This study aimed to clarify the impact of Swanson’s theory of care intervention on the self-efficacy and pregnancy outcomes of primiparous women during pregnancy. The results may provide some reference for optimizing the nursing plan for primiparous women.

## METHODOLOGY

This single-center retrospective case-control analysis retrospectively assessed primiparous women who underwent prenatal check-ups and completed delivery at Suzhou Hospital of Integrated Traditional Chinese and Western Medicine from March 2023 to March 2025. According to the prenatal care received from 28 to 37 weeks of pregnancy, women were divided into the Swanson group (based on Swanson’s theory of care nursing) and the control group (routine nursing). Two groups were matched in a 1:1 ratio based on age, place of residence, education level and the history of abortion or induced labor. Therefore, the following research hypotheses were proposed to guide the study design and statistical analysis: *Hypothesis-1 (H1):* Swanson’s care-based intervention significantly improves self-efficacy in primiparous women, as measured by CBSEI-C32, OE-16, and EE-16 scores; *Hypothesis-2 (H2):* Swanson’s care-based intervention leads to improved childbirth outcomes, including lower labor pain scores, shorter labor duration, higher one-minute Apgar scores, and reduced incidence of cesarean sections and adverse pregnancy outcomes.

### Ethical Approval:

This study has been reviewed and approved by the Suzhou Integrated Traditional and Western Medicine Hospital ethics committee (2025-022); Date: June 12^th^ 2025. Although this was a retrospective study, all participants had signed a written informed consent form upon admission, allowing their clinical data to be used anonymously for future research purposes. Prior to analysis, all patient data were fully de-identified and anonymized to ensure that no personal identifiers were linked to the study dataset. Access to the data was strictly limited to authorized members of the research team, and all data were stored on secure hospital servers in accordance with institutional data protection and confidentiality policies. These procedures ensured that the study was conducted in full compliance with the principles outlined in the Declaration of Helsinki.

### Inclusion criteria:

- Primiparous women.
- Age range: 18-35 years old.
- Individuals without indications for cesarean section.
- Complete clinical data.

### Exclusion criteria:

- Individuals with mental illnesses.
- Patients with major heart, brain, lung, liver and kidney diseases.
- Patients with combined malignant tumors.
- Pregnancy complications such as pregnancy diabetes and pregnancy hypertension.
- Individuals with immune dysfunction disorders.
- Pregnant women under 37 weeks of gestation.

### Outcomes

The primary outcome was self-efficacy and coping ability during the delivery. The Chinese version of the Childbirth Self-Efficacy Inventory (CBSEI-C32) was used for evaluation. CBSEI-C32 was divided into two subscales, Outcome Expectations (OE-16) and Self-Efficacy Expectations (EE-16), each with 16 items. Items were scored on the scale of One to 10, where One represents completely unhelpful/not certain and 10 means very helpful/very certain. Each subscale score ranged from 16 to 160 points. The Chinese version of the CBSEI-C32 used in this study was adapted and psychometrically validated in prior research involving Chinese perinatal populations, and has demonstrated satisfactory internal consistency and construct validity.^25^ However, we acknowledge that cultural interpretations of self-efficacy-such as perceived control over childbirth, pain expression, and expectations of support-may differ across cultural settings, even when a validated translation is used. These cultural nuances may influence how participants perceive and respond to scale items. We have therefore noted this as a potential limitation. Future studies may consider incorporating qualitative interviews or culturally adapted tools to enhance the cultural sensitivity and measurement accuracy of maternal self-efficacy assessments in diverse populations.

The secondary outcomes included the degree of pain during labor, total duration of labor, one minute Apgar score of newborns and adverse pregnancy outcomes. Pain was assessed using a visual analog scale (VAS), with scores ranging from One to 10 (the higher the score, the greater the degree of pain). Adverse pregnancy outcomes included postpartum hemorrhage, postpartum infection, neonatal asphyxia, neonatal and wet lung and overall incidence.

### Nursing approaches:

### Routine care nursing:

It included regular prenatal checkups, pregnancy health education (distributing promotional brochures, centralized teaching), routine care during delivery (monitoring vital signs, observing and guiding the labor process) and postpartum basic care (guidance on neonatal care and postpartum rehabilitation).

### Swanson’s theory of care nursing:

The intervention was delivered in accordance with the five caring processes defined by Swanson’s theory, with operationalization tailored to the perinatal clinical setting. All nursing personnel involved in the intervention received standardized training in Swanson’s model to ensure consistency. The implementation details are as follows:

### Knowing

Conducted during the first prenatal hospital visit (gestational weeks 28-30). Primary nurses conducted in-depth face-to-face interviews to assess the patient’s personal background, psychosocial status, childbirth expectations, and knowledge gaps. Individualized care records were established for each participant.

### Being with:

Maintained through continuous contact during the antenatal period via WeChat messaging and emotional support. During labor, a designated midwife accompanied the participant throughout, offering physical and verbal reassurance (e.g., holding hands, gently touching the forehead, verbal encouragement).

### Doing for:

Implemented during intrapartum and immediate postpartum periods. Responsibilities included maternal safety monitoring, guided breathing, massage for relaxation, and assistance with early postpartum recovery activities such as breastfeeding and hygiene management. 

### Enabling

Delivered between gestational weeks 30-32 by trained nurse educators through structured scenario simulations, instructional videos, and small-group sessions. Content covered delivery preparation, coping skills, and postpartum self-care education.

### Maintaining belief:

Conducted during home-based postpartum follow-ups (within 1-2 weeks after discharge) by community or hospital-affiliated postpartum nurses. Support was provided to enhance maternal confidence, address emotional concerns, and promote maternal-infant bonding through structured counseling.

### Statistical Analysis:

The Statistical Package for Social Sciences Statistics 26.0 (IBM, NY, USA) was used to input, examine and analyze data collected from medical records and questionnaires. In this study, categorical variables (such as the pregnant woman’s place of residence, education level, school education, experience of abortion or induced labor, mode of delivery, occurrence of adverse pregnancy outcomes, etc.) were expressed as frequency and proportion and chi square tests were used to compare the differences between the two groups.

The Shapiro-Wilk test was used to assess the normality of the continuous variable distributions. For normally distributed data, mean ± standard deviation was used to represent normal distribution data. Independent sample t-test is used for intergroup comparisons and paired t-test is used for intra-group comparisons, such as comparing OE-16, EE-16 and CBSEI-C32 scores at baseline and after intervention. Non-normally distributed data were represented by median and interquartile range (IQR) and the Mann-Whitney U test was used for intergroup comparisons, such as age, delivery VAS, total duration of labor and neonatal one minute Apgar score. All P-values were double-tailed and P<0.05 was considered statistically significant.

## RESULTS

**Fig.1:**
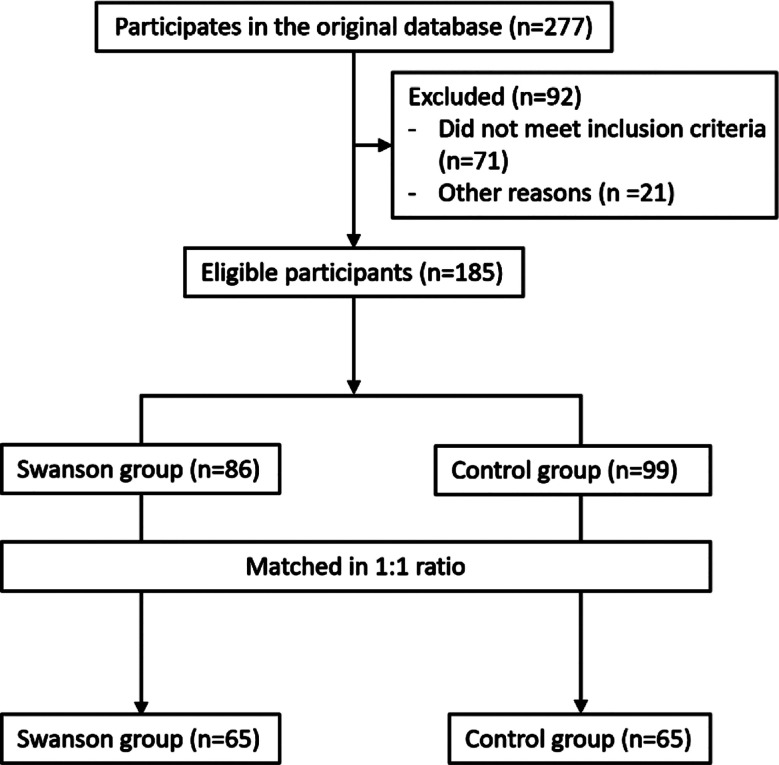
Screening Process for Two Groups of Patients.

In this single-center, retrospective case-control analysis, a total of 185 primiparous women met the eligibility criteria. Of them, 130 primiparous women (age range 19-35 years, median age 27 (25, 32) years) were matched in a 1:1 ratio, with 65 women in the Swanson group and 65 in the control group (Fig.1). There was no significant difference in basic information such as age, place of residence, education level, maternal school education and experience of abortion or induced labor between the two groups of patients (P>0.05) (Table-I).

**Table-I:** Comparison of basic information between Swanson group and control group.

Basic information	Swanson group (n=65)	Control group (n=65)	Z/χ^2^	P
Age (years), M(IQR)	26 (24, 31)	28 (25, 32)	-1.169	0.242
Place of residence, n(%)			0.381	0.537
Town	48 (73.8)	51 (78.5)		
Rural area	17 (26.2)	14 (21.5)		
Educational level, n(%)			0.615	0.433
Junior high school and below	20 (30.8)	16 (24.6)		
High school and above	45 (69.2)	49 (75.4)		
Pregnant women’s school learning, n(%)	9 (13.8)	5 (7.7)	1.281	0.258
Experience of miscarriage or induced abortion (time), n(%)			3.602	0.165
0	55 (84.6)	45 (70.8)		
1	7 (10.8)	13 (20.0)		
≥2	3 (4.6)	6 (9.2)		

Before intervention, both groups had comparable EE-16, OE-16 and CBSEI-C32 scores (P>0.05). After intervention, the EE-16 (120.2 ± 21.2 vs. 106.6 ± 24.1), OE-16 (115.4 ± 21.0 vs. 103.3 ± 23.5) and CBSEI-C32 (235.6 ± 33.7 vs. 209.9 ± 40.2) scores in both groups significantly increased compared to before intervention and were considerably higher in the Swanson group (P<0.05) (Table-II).

**Table-II:** Comparison of EE-16, OE-16 and CBSEI-C32 scores between two groups.

Index	Swanson group (n=60)	Control group (n=60)	t	P
***Baseline***				
OE-16	90.5±25.5	94.8±23.5	-1.006	0.317
EE-16	88.0±23.3	93.8±27.9	-1.298	0.197
CBSEI-C32	178.5±46.6	188.7±48.5	-1.220	0.225
***After intervention***				
OE-16	120.2±21.2#	106.6±24.1#	3.426	0.001
EE-16	115.4±21.0#	103.3±23.5#	3.103	0.002
CBSEI-C32	235.6±33.7#	209.9±40.2#	3.959	<0.001

***Note:*** Compared to baseline,

# P<0.05.

Compared to the control group, the Swanson group had significantly lower delivery VAS score (5 (5,6) vs. 6 (5,8)), total labor duration (7 (5,8) vs. 9 (8,10)), cesarean section rate (8 (12.3) vs. 17 (26.2)) and incidence of adverse outcomes (10 (15.4) vs. 20 (30.8)), while the one minute Apgar score (9 (9, 10) vs. 9 (9, 9)) of newborns was significantly higher than that in the control group (P<0.05) (Table-III).

**Table-III:** Comparison of perinatal indicators and adverse pregnancy outcomes between two groups.

Indicator	Swanson group (n=60)	Control group (n=60)	χ2/t	P
Delivery VAS score, M(IQR)	5 (5, 6)	6 (5, 8)	-2.954	0.003
Total labor duration, M(IQR)	7 (5, 8)	9 (8, 10)	-4.590	<0.001
1-minute Apgar score, M(IQR)	9 (9, 10)	9 (9, 9)	-2.579	0.010
***Cesarean section, n(%)***				
Caesarean section	8 (12.3)	17 (26.2)	1.207	0.230
Natural childbirth	57 (87.7)	48 (73.8)	4.011	0.045
Incidence of adverse outcomes, n(%)	10 (15.4)	20 (30.8)	4.333	0.037
Postpartum hemorrhage, n(%)	3 (4.6)	4 (6.2)		
Puerperal infection, n(%)	4 (6.2)	4 (6.2)		
Neonatal asphyxia, n(%)	2 (3.1)	7 (10.8)		
Neonatal wet lung, n(%)	1 (1.5)	2 (3.1)		
Two or more adverse outcomes, n(%)	0 (0.0)	3 (4.6)		

## DISCUSSION

This study demonstrated that compared to the traditional nursing approach, Swanson’s care theory nursing can more effectively enhance the self-efficacy of primiparous women during labor, reduce their pain levels, shorten labor duration and is associated with increased vaginal delivery rates, as well as improved maternal and infant outcomes.

Swanson’s theory of care approach was associated with significantly higher scores on self-efficacy-related scales such as EE-16, OE-16 and CBSEI-C32 compared to the routine nursing intervention. The results of this study are consistent with previous reports.^26^-^28^ Fenwick et al.^26^ found that a brief antenatal midwife-led psychological education intervention on fear of childbirth can help reduce maternal anxiety and the incidence of cesarean section and promotes vaginal births in subsequent pregnancies. Wu et al.^27^ confirmed that mindfulness-based psychological interventions have a positive impact on improving the self-efficacy and emotional health of elderly primiparous women with postpartum depression and on pregnancy outcomes. In addition, Ortega Barco et al.^28^ implemented nursing based on Swanson’s theory during childbirth and demonstrated that it can significantly improve the satisfaction and experience of pregnant women.

This study demonstrated higher advantages of Swanson-based care in first-time mothers. It is possible that, based on the Swanson’s care theory, interventions for primiparous women from a more systematic and comprehensive perspective not only include psychological support and health education, but also emphasize the importance of companionship, empowerment and assistance in the growth of primiparous women.^27^ Compared to traditional nursing, the theoretical system of Swanson’s care theory is more comprehensive and intervention measures are more targeted and operable.

In clinical obstetric nursing, a reduction of 1 to 2 points on the VAS pain scale is widely regarded as clinically meaningful. This degree of pain relief can reduce the need for pharmacological analgesia, enhance maternal cooperation during labor, and improve the overall childbirth experience.^28^ Similarly, a decrease of 1 to 2 hours in total labor duration may reduce maternal fatigue, lower the risk of uterine inertia, and improve neonatal outcomes by reducing the likelihood of fetal distress.^29^ These results indicate that the statistically significant differences observed in this study also hold substantial clinical value. The Swanson-based intervention therefore offers not only theoretical and psychological benefits, but also practical improvements that can be directly translated into routine nursing care protocols.

The results of this study showed that the Swanson group had significantly lower delivery VAS scores and total labor duration compared to the control group. This result suggests that interventions based on Swanson’s care theory can enhance the self-efficacy of postpartum women, enabling them to approach various childbirth conditions with optimism and composure and improve their understanding of pain management, ultimately reducing labor time. This is consistent with the research results of Çankaya et al.^29^ and Duncan et al.^30^ It is plausible that the psychological comfort provided to mothers throughout the delivery process makes them more optimistic and confident, thereby reducing the increase in catecholamine secretion caused by excessive stress and, consequently, reducing the risk of uterine contractions and prolonged labor.^28^-^30^ Indeed, according to the study of prenatal delivery video tutorials, improved self-efficacy promotes the smooth progress of the labor process.^30^ This effect may be explained by the psychophysiological mechanisms involving the hypothalamic-pituitary-adrenal (HPA) axis. Psychological stress and anxiety can activate the HPA axis, increasing the release of catecholamines such as adrenaline and noradrenaline. Elevated levels of these stress hormones have been shown to inhibit the release of oxytocin-a key hormone responsible for initiating and maintaining effective uterine contractions.^30^ As a result, excessive stress may lead to uterine dysfunction, longer labor, and increased delivery-related complications. Conversely, the emotional support, empowerment, and belief-maintenance components emphasized in Swanson’s theory may help reduce maternal anxiety, attenuate the stress response, and facilitate the secretion of endogenous oxytocin, thereby promoting coordinated uterine contractions and shortening the labor process.^31^

To further understand the reduction in adverse pregnancy outcomes observed in this study, several potential mechanisms related to the Swanson care-based intervention can be considered. Firstly, the continuous emotional support provided by trained nurses and midwives may alleviate psychological stress and reduce maternal anxiety levels, thereby lowering excessive catecholamine secretion. This helps maintain stable uteroplacental perfusion, improves fetal oxygenation, and reduces the risk of neonatal asphyxia.^32^ Secondly, the enhanced prenatal education and postpartum behavioral guidance in the intervention group likely contributed to improved hygiene practices, adherence to early mobilization, and effective breastfeeding-each of which is critical for minimizing the risk of postpartum infection and complications.^33^ Lastly, the incorporation of individualized coping strategies, such as guided breathing and relaxation training, may have promoted more efficient uterine contractions and reduced labor fatigue, thereby preventing prolonged labor and associated risks.^34^ These multifaceted pathways provide plausible explanations for the observed improvements in maternal and neonatal outcomes.

The results of this study showed that the 1-minute Apgar scores of newborns in the Swanson group were significantly higher than those in the control group. This effect may be due to the ability of Swanson’s care approach to stabilize women’s emotions, lower the stress response level and ensure that the fetus receives a sufficient oxygen and nutrition supply, thereby reducing the risk of fetal distress and ensuring the newborn’s health status at birth.^34^ Beyond the immediate clinical benefits observed in this study, Swanson’s theory of caring holds significant potential for enhancing the broader clinical nursing system. First, in obstetric nursing education, the five-process model (knowing, being with, doing for, enabling, and maintaining belief) provides a structured framework for teaching humanistic care principles, empathy development, and individualized nursing interventions.^35^ Second, in emotional management, the relational and presence-based aspects of the model can be integrated into standardized pathways for addressing prenatal anxiety and postpartum mood disorders.^36^ Finally, for postpartum rehabilitation, the concepts of continuity of care and belief support lend themselves well to home follow-ups, breastfeeding guidance, and maternal-infant bonding programs.^37^ Taken together, these applications suggest that Swanson’s theory may serve not only as an effective care model but also as a foundational framework for shifting obstetric nursing systems from task-centered to person-centered paradigms. In terms of pregnancy outcomes, the cesarean section rate and overall incidence of adverse pregnancy outcomes in the Swanson group were significantly lower than those in the control group. Swanson’s care theory enhances the self-efficacy of primiparous women in terms of psychology, knowledge and skills, making them more confident in natural childbirth and reducing the risk of postpartum hemorrhage and adverse neonatal outcomes.^37^ Guo et al.^37^ demonstrated that effective intervention measures can reduce adverse physiological and psychological reactions in primiparous women, improve natural delivery rates and postoperative recovery, decrease the risk of neonatal asphyxia and ensure the safety of both mothers and infants during the perinatal period.

While Swanson’s theory of caring offers a structured and individualized framework for maternal care, we acknowledge that its real-world implementation-particularly in resource-limited primary care settings-may face practical constraints. First, the model emphasizes continuous emotional presence and individualized attention, which can be difficult to sustain in clinical environments with limited nursing staff. Second, the time demands of implementing all five caring processes may exceed the capacity of nurses in high-volume obstetric units. Third, ensuring fidelity to the model requires structured training programs and periodic refreshers, which may not always be feasible in under-resourced facilities. To address these challenges, we suggest that future adaptations of Swanson’s model consider modular implementation (prioritizing certain components based on clinical capacity), group-based education formats, and the use of digital platforms-such as mobile apps, video tutorials, or chatbot-assisted counseling-to deliver scalable, hybrid interventions. These strategies may enhance the model’s accessibility and operational feasibility across diverse healthcare tiers.

### Strength of this study:

First, it was guided by a well-established theoretical framework-Swanson’s theory of caring-which provides a coherent conceptual structure for nursing interventions. Second, the intervention was implemented in a real-world clinical setting using a structured, replicable protocol, enhancing its practical relevance and reproducibility. Third, the study evaluated both psychological (maternal self-efficacy) and obstetric (labor-related) outcomes, providing a comprehensive understanding of the intervention’s effects. However, several areas warrant further investigation. Future research should include larger sample sizes and utilize multi-center randomized controlled trials (RCTs) to strengthen causal inference. Longitudinal follow-up is needed to assess sustained outcomes such as postpartum depression, breastfeeding continuation, and maternal-infant bonding. Furthermore, adaptations of Swanson-based interventions should be tested in other populations, such as high-risk pregnancies, multiparous women, and resource-constrained healthcare settings, to determine broader applicability and scalability.

In summary, this study confirms that interventions based on Swanson’s care theory can improve self-efficacy and pregnancy outcomes in primiparous women. To our knowledge, this is the first study to observe the effects of intervention measures based on Swanson’s care theory on self-efficacy and pregnancy outcomes in this population.

### Limitations of the study:

This study has several limitations that should be acknowledged. First, it was a retrospective, single-center case-control study with a relatively small sample size (n = 130), which limits the generalizability and statistical power of the findings. Although a 1:1 matching strategy was applied based on key baseline characteristics (e.g., age, residence, education level, and obstetric history), and appropriate statistical controls were used, the design inherently carries risks of selection bias and residual confounding. Consequently, causal interpretations between the intervention and outcomes should be made with caution. Second, the study excluded primiparous women over the age of 35 and those with gestational age less than 37 weeks, further narrowing the applicability of the results to broader maternal populations, such as high-risk or multiparous women. Third, while the intervention appeared to have short-term benefits on self-efficacy and perinatal outcomes, long-term postpartum effects-such as maternal physical recovery, breastfeeding continuation, psychological well-being, and maternal-infant bonding-were not assessed due to the lack of longitudinal follow-up data in the clinical records.

Fourth, maternal mental health indicators, including prenatal anxiety and postpartum depression, were not evaluated because standardized psychological assessments (e.g., GAD-7, EPDS, SDS) were not routinely documented in the retrospective dataset. To address these limitations, we plan to conduct a large-scale, multi-center prospective randomized controlled trial (RCT) with predefined sample size calculations and structured postpartum follow-up at multiple time points (e.g., six weeks, three months, and six months). This will allow for more robust evaluation of both short- and long-term effects of Swanson’s theory-based interventions, including their impact on maternal psychological outcomes. Notably, Swanson’s caring model-particularly the elements of “being with” and “maintaining belief”-emphasizes emotional support, which may contribute positively to maternal mental health and warrants further investigation.

## CONCLUSION

The findings of this study suggest that nursing interventions based on Swanson’s theory of caring can effectively enhance maternal self-efficacy, alleviate labor pain, shorten the duration of labor, reduce the incidence of cesarean section and adverse pregnancy outcomes, and overall improve childbirth outcomes among primiparous women. This theory-guided, structured, and psychologically-informed approach offers a scientific and replicable framework for the care of first-time mothers, integrating emotional support, individualized care, and cognitive empowerment. However, it is important to note that the study population consisted exclusively of primiparous women, which limits the generalizability of the findings. While the core principles of Swanson’s model may also be beneficial for multiparous or high-risk pregnant women, further research is warranted to examine its adaptability, effectiveness, and implementation strategies in broader and more diverse maternal populations. High-quality, multi-center prospective studies with long-term follow-up will be essential to further validate the effectiveness and scalability of Swanson’s care-based interventions in clinical practice.

### Authors’ contributions:

**CZ:** Literature search, study design and manuscript writing.

**CZ and YZ:** Data collection, data analysis and interpretation. Critical review.

**CZ:** Manuscript revision and validation and is responsible for the integrity of the study.

All authors have read and approved the final manuscript.
